# Antioxidants of Natural Products

**DOI:** 10.3390/antiox10040612

**Published:** 2021-04-16

**Authors:** Mee Ree Kim

**Affiliations:** Department of Food and Nutrition, Chungnam National University, Daejeon 34134, Korea; mrkim@cnu.ac.kr

Antioxidant ingredients are known to contribute to the beneficial effects of natural products in health promotion as well as disease prevention by reducing oxidative stress, caused by reactive oxygen or nitrogen species, in biological systems [1]. Various antioxidants of natural products demonstrate pharmacological actions such as anti-inflammatory, anticancer, cardioprotective, neuroprotective, antiaging, etc. Recent studies have been carried out to optimize the pharmacological action of antioxidants derived from edible plants, herbs, spices or seaweeds [1,2]. Moreover, natural products from edible plants, compared with synthetic antioxidants, are considered to be more acceptable and reliable, since humans are accustomed to the use of those plant extracts [3]. Further, the use of phytomedicine containing antioxidants has been increased due to the efficacy and low cost in primary care setting of clinical practice. This Special Issue covers the following aspects; the new insight into the pharmacological action of extracts containing antioxidant constituents, the formulation of antioxidants for maximal activity or bioavailability, and the practical aspect of the specified antioxidants for clinical application.

First, this issue provides a new insight into the biological action of herbal extracts containing antioxidants. The polyphenol-rich extract of *Ribes dicanthum* exerted a neuroprotective action through BDNF/TrkB pathway in glutamate-stimulated HT22 cells, and prevented against scopolamine-induced amnesia [4]. A similar action was also observed with the extract of *Enteromorpha prolifera,* a green algae [5]. Additionally, the extract of *Ishie okamurae*, belonging to a brown algae, expressed a neuroprotective action by regulating the MAPKs/Nrf-2/HO-1 pathway [6]. Likewise, the extract of *Euonymus alatus* alleviated the scopolamine-induced memory deficit through BDNF-mediated activation of Nrf2 signaling [7]. Further, the extract of *Platycodon grandiflorum* root protected against Aβ-induced cognitive dysfunction and pathology in female models of Alzheimer’s disease [8]. Besides, the extract from new cultivars of sweet cherry(*Prunus avium* L.) containing cyanidine-3-O-rutinoside as a major anthocyanin, where the antioxidant capacity was positively correlated with total anthocyanin index, demonstrated an antioxidant/neuroprotective action through downregulation of oxidative stress and upregulation of BDNF in neuronlike SH-Sy5Y cells [9]. Taken together, these plant extracts could be used as phytomedicine, which protects against neurodegenerative disorders such as Alzheimer’s disease, etc.

Separately, the extract of *Ribes dicanthum* exerted anti-inflammatory action through the upregulation of Nrf2/HO-1 and the downregulation of *NF-kB* signaling in LPS-stimulated RAW 264.7 cells [10]. Similarly, *Azolla pinnate* extract containing polyphenols, demonstrated anti-inflammatory and antiapoptotic action, contributing to the curative action against lead-induced hepatotoxicity [11]. In addition, Geno TX-407, the combination of *Scutellaria baicalensis* extract and *Magnolia officinalis* extract, designed for pharmacological advantage, exerted greater anti-inflammatory action than each single extract via the Nrf2/HO-1 and NF-kB signaling pathway [12]. It is noteworthy that the extract of *Lycium barbarum* leaf containing polyphenols, which strengthened a tight junction integrity and reduced NO production, suppressed ER stress and LPS-induced inflammation via the IRE1 alpha and XBP1 pathway in Caco-2 cells as well as in inflamed intestine of mice [13]. Similarly, black ginseng (*Panax Ginseng*) extract containing Rb1, Rg3 and Rk1 was observed to express antioxidant and anti-inflammatory action in an IRE1 alpha-dependent and XBP1-independent manner in the ER stress pathway [14]. Besides, the extract of *Cephalaria gigantea* and *Cephalaria uralensis*, containing 5-O-caffeoylquinic acid, isoorinetin and swertiajaponin, expressed potential action as an antiacne agent by showing anti-inflammatory action and antibacterial activity against S aureus, S epidermidis and P aces [15], and the extract of black soybean (*Yak-Kong*), fermented by lactic acid bacteria, and expressed an anti-aging effect by markedly reversing sUV-induced matrix metalloproteinase-1 in human keratinocytes [16]. Thus, antioxidant constituents from natural sources have been continuously explored in an attempt to find practical applications for phytomedicines. Consonant with this, the polyphenols in the extract of *Vitis vinifera* L. leaf were suggested to protect against various oxidative stress-induced disorders [17]. In an independent study to elucidate the biological action of a single antioxidant compound, xanthohumol, a major prenylated chalcone in hops alleviated vascular calcification in the model of Vit D3/nicotine-induced vascular calcification through the activation of the Nrf2//Keap1/HO-1 pathway [18]. In addition, acteoside, a major antioxidant of *Abeliophyllum distichum* extract was proposed to be an effective antioxidant as a functional supplement [19].

Next, the formulation of antioxidants, such as antioxidant conjugates or fatty acyl hybrid of antioxidants, may contribute to the increase in the bioactivity or bioavailability in vivo system. Gallic acid conjugates were evaluated for the increase in antioxidant action; gallic acid–laminarin conjugate, more potent as than laminarin or carboxylated laminarin, protected against H_2_O_2_-induced oxidative damage in MDCK cells, suggesting a potential application of the modified antioxidants in functional supplements [20]. In a separate study, the bioavailability of the antioxidant was elucidated using the oleoyl hybrid of the antioxidant. The regioisomers of oleoyl-hybrids of quercetin, which were prepared to overcome low bioaccessibility limitations, exhibited a highly selective anticancer cytotoxicity and greater cytotoxic action than parental compounds [21].

Lastly, the review on specified antioxidants may expand the knowledge about the practical applications of antioxidants. Thymoquinone, a chief active constituent of *Nigella sativa*, is known to have various bioactivities. In particular, the potential role of thymoquinone or *Nigella sativa* extract as an adjuvant in periodontal therapy was proposed [22]. Nonetheless, further studies are required to establish the clinical efficacy and safety of thymoquinone or the extract. Separately, the antioxidant action of ellagic acid and its metabolite, urolithin, was discussed in respect to the bioavailability [23]. It is of note that urolithin reached the target tissue to a greater extent than ellagic acid, thus appearing as the main metabolite responsible for the beneficial action of ellagic acid. However, the formation of urolithin greatly depends on the metabotype of an individual, limiting its potential as a therapeutic agent. Therefore, the application of ellagic acid or urolithin needs further pharmacokinetic and clinical studies.

Here, the pharmacological action of antioxidants from natural sources or extracts containing antioxidant constituents was reviewed, with the mode of action being highlighted. However, the toxicological aspects of the antioxidant compounds and the extracts containing antioxidants need further studies for their practical application. Additionally, the stability of the antioxidants or the extract preparation must be guaranteed for their convenient use and economic benefit.
